# Induction of Treg and alternatively activated macrophages by the helminth *Echinococcus granulosus*: implication in the promotion or control of allergic disease?

**DOI:** 10.1186/2045-7022-4-S2-P61

**Published:** 2014-03-17

**Authors:** Manel Amri, Chafia Touil-Boukoffa

**Affiliations:** 1 University of Sciences and Technology Houari Boumediene, Department of Celluar and Molecular Biology, Algiers, Algeria

## Background

The effect of helminth infections on allergic diseases is still inconclusive. Several studies show that Treg cell induced by parasites down regulate the host Th2-mediated allergic response. However, some studies highlight the importance of alternative activated macrophages (AAM) in the pathogenesis of allergic diseases. This phenotype is also implicated in helminth survival. Our aim is to study the immunomodulatory effect of laminated layer (LL, accelullar layer of hydatic cyst) of the helminth *Echinococcus granulosus*. This infection remains a serious parasitic disease in Algeria.

## Method

We have investigated the effect of LL extract (LLs) on IL-10 and IFN-γ production by human mononuclear cells (PBMC) *in vitro*. Moreover, the effect of LLs on macrophages phenotype and expression of some M1/M2 markers was investigated. NOS2 and Arginase activities were also evaluated. Moreover, implication of mannose receptor (MR) and TGF-β on Arginase activity was evaluated using mannose (MR antagonist) and Anti-TGFβ. Activity of NADPH oxidase was also evaluated by chemiluminescence. Finally, TLR2, TLR4, CD14 and CD23 expression was evaluated by flow cytometric immunoassay.

## Results

We have found that LLs enhanced IL-10 production and reduced IFN-γ production. Moreover, while NOS2 and NADPH oxidase activities are inhibited, Arginase activity is stimulated. Interestingly, MR and TGF-β seem to be implicated in the Arginase induction by LLs. Moreover, LLs increases TLR2 and CD14 expression and decreases CD23 expression by monocytes.

## Conclusion

Our data support the hypothesis that helminth infections elicit a Treg population able to down- regulate allergen induced allergic diseases. The implication of TGF-β in the induction of AAMs phenotype supports our finding of a concomitant activation of Treg and AAMs populations by LL. The potential therapeutic or promoting effect of Laminated Layer in allergic diseases remains to be investigated in mouse model of allergic diseases.

